# Supplementary material for: FDAAA legislation is working, but methodological flaws undermine the reliability of clinical trials: a cross-sectional study
Source: PeerJ. 2015 Jun 25;3:e1015. doi: 10.7717/peerj.1015 (PMC4485238; doi:10.7717/peerj.1015)
Supplement: Appendix S1 [file peerj-03-1015-s001.pdf]

APPENDIX 1. SEARCH STRATEGY FOR PROTOCOLS REGISTERED ON CLINICALTRIALS.GOV

Sample

Convenience sampling (every protocol that met the inclusion criteria).

Inclusion criteria

(a) Phases III or IV clinical trials;
(b) Closed clinical trials;[I]
(c) Interventional trials;[II] and
(d) Registered trials of adalimumab, bevacizumab, rituximab, trastuzumab, or infliximab, regardless of location or language.

Exclusion criteria

We excluded protocols in which adalimumab, bevacizumab, rituximab, trastuzumab, or infliximab were not set as the primary test or the primary control drug. We also excluded protocols not completed as of May 8, 2012 (our search date at www.clinicaltrials.gov).

Entry terms

We searched for protocols of clinical trials registered on ClinicalTrials.gov in accordance with the following Medical Subject Headings (MeSH terms).[III]

Table 1. Search Strategy: MeSH Terms

Name of biologic	*MeSH Terms*
Adalimumab	D2E7 antibody; Humira; Adalimumab; LU200134
Bevacizumab	Avastin; Bevacizumab
Infliximab	Monoclonal antibody cA2; MAb cA2; Remicade; Infliximab
Rituximab	CD20 antibody; Rituximab; Mabthera; Rituxan; IDEC C2B8 antibody; IDEC C2B8
Trastuzumab	Trastuzumab; Herceptin

We evaluated each protocol individually and completed a form with the following information: (a) title of the study, (b) condition,[IV] (c) study phase,[V] (d) NCT number,[VI] (e) intervention,[VII] (f) sponsor, (g) funded by (…), (h) starting date, (i) completion date, (j) sites/location (U.S. or non-U.S.), (k) randomized allocation (Yes or No),[VIII] (l) single arm/single group design (Yes or No),[IX] (m) masking,[X] (n) reporting of results (Yes or No),[XI] and (o) responsible party.

[I] "Clinical studies that are no longer recruiting participants because they have enough participants already, because they are completed, or because they have been stopped for some reason. This also describes studies with very specific eligibility criteria that recruit participants by invitation only." Available at http://clinicaltrials.gov/ct2/about-studies/glossary.

[II] "A clinical study in which participants are assigned to receive one or more interventions (or no intervention) so that researchers can evaluate the effects of the interventions on biomedical or health-related outcomes. The assignments are determined by the study protocol. Participants may receive diagnostic, therapeutic, or other types of interventions." Available at http://clinicaltrials.gov/ct2/about-studies/glossary.

[III] "MeSH is the National Library of Medicine's controlled vocabulary thesaurus. It consists of sets of terms naming descriptors in a hierarchical structure that permits searching at various levels of specificity." Available at http://www.nlm.nih.gov/pubs/factsheets/mesh.html.

[IV] According to the ClinicalTrials.gov website, the term condition refers to a "disease, disorder, syndrome, illness, or injury that is being studied. In ClinicalTrials.gov, conditions may also include other health-related issues such as lifespan, quality of life, and health risks." Available at http://clinicaltrials.gov/ct2/about-studies/glossary.

[V] "Food and Drug Administration (FDA) categories … for describing the clinical trial of a drug based on the study's characteristics, such as the objective and number of participants. There are five phases:
Phase 0: Exploratory study involving very limited human exposure to the drug, with no therapeutic or diagnostic goals (for example, screening studies, microdose studies).
Phase 1: Studies that are usually conducted with healthy volunteers and that emphasize safety. The goal is to find out what the drug's most frequent and serious adverse events are and, often, how the drug is metabolized and excreted.
Phase 2: Studies that gather preliminary data on effectiveness (whether the drug works in people who have a certain disease or condition). For example, participants receiving the drug may be compared with similar participants receiving a different treatment, usually an inactive substance (called a placebo) or a different drug. Safety continues to be evaluated, and short-term adverse events are studied.
Phase 3: Studies that gather more information about safety and effectiveness by studying different populations and different dosages and by using the drug in combination with other drugs.
Phase 4: Studies occurring after FDA has approved a drug for marketing. These including postmarket requirement and commitment studies that are required of or agreed to by the sponsor. These studies gather additional information about a drug's safety, efficacy, or optimal use." Available at http://clinicaltrials.gov/ct2/about-studies/glossary.

[VI] "A unique identification code given to each clinical study registered on ClinicalTrials.gov. The format is the letters 'NCT' followed by an 8-digit number (for example, NCT00000419)." Available at http://clinicaltrials.gov/ct2/about-studies/glossary.

[VII] "A process or action that is the focus of a clinical study. This can include giving participants drugs, medical devices, procedures, vaccines, and other products that are either investigational or already available. Interventions can also include noninvasive approaches such as surveys, education, and interviews." Available at http://clinicaltrials.gov/ct2/about-studies/glossary.

[VIII] "A strategy in which participants are assigned to arms of a clinical trial by chance." Available at http://clinicaltrials.gov/ct2/about-studies/glossary.

[IX] "Describes a clinical trial in which all participants receive the same intervention." Available at http://clinicaltrials.gov/ct2/about-studies/glossary.

[X] "A clinical trial design strategy in which one or more parties involved with the trial, such as the investigator or participant, do not know which participants have been assigned which interventions.

Types of masking include none, Open label, Single blind masking, and Double blind masking." Available at http://clinicaltrials.gov/ct2/about-studies/glossary.

[XI] "The process of submitting and updating summary information about the results of a clinical study to a structured, public Web-based results database, such as the ClinicalTrials.gov results database." Available at http://clinicaltrials.gov/ct2/about-studies/glossary.
