# Supplementary material for: FDAAA legislation is working, but methodological flaws undermine the reliability of clinical trials: a cross-sectional study
Source: PeerJ. 2015 Jun 25;3:e1015. doi: 10.7717/peerj.1015 (PMC4485238; doi:10.7717/peerj.1015)
Supplement: Appendix S2 [file peerj-03-1015-s002.pdf]

APPENDIX 2. SEARCH STRATEGY FOR PUBLISHED PAPERS

Based on the data gathered from ClinicalTrials.gov, we searched for published trials on Pubmed, Embase, Lilacs, Cochrane Central, and Google Scholar according to the following search strategy:

(A) Search for the National Clinical Trials (NCT) number;
(B) Search for the name of the primary investigator;
(C) Search for the name of other investigators;
(D) Search for MeSH descriptors of the biological agents (see Appendix 1); and
(F) Search for the title of the protocol.

Studies were considered *published* when a scientific paper or an abstract was published in an indexed journal or in conference proceedings.
